# "Progress" and the Hospital Pageant

**Published:** 1921-08-20

**Authors:** 


### "Progress" and the Hospital Pageant.

With the August number, *Progress*, the journal of the Great Northern Central Hospital, begins its second year. It is already distinguished among journals published by institutions for its wider outlook, its interest in other hospitals' doings, its endeavour to be generally informing. The paper has also tried to reach those "who, in a general way, would be out of touch." The current number includes an account of the recent meeting of hospital secretaries to discuss the idea of a Hospital Week and Pageant, and Lord Burnham's recent speech in the House of Lords on Hospital Finance and the Approved Societies' Surplus. Lord Burnham has lately become a Vice-President of the hospital. Now, it will be agreed that the meeting to discuss the holding of a Hospital Week and Pageant and the above speech are of general interest, and yet the kind of subject hitherto excluded, almost on principle, from hospital gazettes and magazines. It seems to us, now that the number of these hospital papers is growing, that they can do good service by giving publicity to such matters, and that, even if their circulation cannot be general, they will yet interest a class of persons which in other ways is beyond the influence of a hospital. Another article in this number is "A Visit to Belgium," by Miss F. Marie Imandt, and a correspondence is reprinted from the *British Medical Journal* concerning the scheme for "contributors' certificates" at the Great Northern Central Hospital, wherein Mr. G. G. Panter distinguishes between these certificates and the old and evil recommendations or subscribers' letters. A good number.

